# Understanding Support for Municipal Political Parties: Evidence from Canada

**DOI:** 10.1177/10780874231224707

**Published:** 2024-01-15

**Authors:** Michael McGregor, Jack Lucas, Chris Erl, Cameron D. Anderson

**Affiliations:** 1 Politics and Public Administration, 7984 Toronto Metropolitan University, Toronto, Ontario, Canada; 2 Political Science, University of Calgary, Calgary, Alberta, Canada; 3 Political Science, University of Western Ontario, London, Ontario, Canada

**Keywords:** municipal elections, political parties, public opinion, Canada

## Abstract

The province of Ontario, Canada, has a longstanding history of non-partisanship in municipal elections. In this distinctive context, we report results on citizen attitudes toward municipal partisanship using a survey of eligible voters in Canada's most populous province. Using a mixed-methods approach, we focus on three interrelated research questions. First, how much does citizen support for municipal parties depend on the *type* of party under consideration? Second, what reasons do citizens provide for their preference for either municipal political parties or independents? Finally, what are the correlates of support for municipal parties? We find little support for municipal political parties, and that many voters have sophisticated reasons for preferring either independents or parties. We also identify several factors associated with support for parties. These results provide an in-depth picture of attitudes on municipal partisanship in Ontario, and suggest that public opinion may provide an overlooked mechanism that maintains Ontario's non-partisanship.

## Introduction

Political parties are active participants in municipal elections across the globe, from South Korea and Iceland to the United Kingdom and Brazil (Boulding and Brown 2015; Copus 2004; Gnarr, 2014; Yoon and Shin 2017). Their presence in local elections is commonplace and accepted among electorates in much of the world. Non-partisan local politics is less common, and is found only in select locations, primarily in Australia, New Zealand/Aotearoa, the western United States, and in most of Anglophone Canada (Webster et al. 2019). In these contexts, non-partisanship at the local level is often considered a by-product of the “Reform Era,” during which time business-oriented urban progressives sought to curb the power of parties and the immigrant communities from which they drew their support. High-profile instances of party-backed corruption in the United States were used across the Anglosphere as evidence that partisanship in municipal affairs brought graft, greed, and disorder (Hawley 1973).

Few jurisdictions have demonstrated as consistent a commitment to municipal non-partisanship as Ontario, Canada. Ontario – Canada's largest province, with more than 14 million residents, 444 municipalities, and some 3,200 distinct municipal positions – holds municipal elections every four years in which candidates seek office as independents, with no political parties listed on the ballot. Even *informal* partisanship is generally absent: municipal candidates largely refrain from advertising whatever party affiliation they may have from other levels of government (many have no personal party affiliation at all), and unless a candidate happens to have had a previous provincial or federal political career, residents often know nothing about the party affiliation of the candidates they are being asked to consider when they cast their municipal votes. While voters do make assumptions about candidates’ party affiliations, these assumptions are often incorrect, and even when a candidate's party affiliation should be obvious (e.g., a former provincial or federal politician), many municipal residents still do not make the link between candidates and their parties (McGregor, Moore, and Stephenson 2016; Lucas and Santos 2022).

This longstanding commitment to municipal non-partisanship in Ontario is often taken for granted by researchers and policymakers. Yet there is much that is noteworthy about Ontario's municipal non-partisanship. For starters, municipal candidates *do* seek office under party banners in some other provinces, and in many other advanced democracies, partisan municipal elections are the norm rather than the exception. Moreover, evidence from analyses of municipal vote choice and council roll call data in Canadian cities suggests that municipal elections and policy debates feature a recognizably low-dimensional structure, closely related to the left-right ideology that animates Canadian politics at other levels of government, making political parties a potentially useful informational and policy-making tool for local candidates (Lucas and McGregor 2021; Lucas, McGregor, and Bridgman 2023; Eidelman and Lucas 2023). Common concerns about political accountability, clarity of responsibility, and municipal turnout in Ontario^1^ – all of which would arguably be improved by party competition – add to the puzzling absence of local parties.

Of the few explanations that have been offered for Ontario's non-partisanship, perhaps the most common has to do with incumbent incentives: on this view, the municipal sector has fallen into an equilibrium in which municipal incumbents have no incentive to encourage or join political parties or slates in local elections (Sayers and Lucas 2017). When incumbents enjoy re-election rates in the 80–90 percent range, the argument goes, these candidates have every reason to discourage institutional innovation that might disrupt the status quo. But this argument overlooks the obvious fact that municipal *challengers* may still find value in creating local parties and slates even if municipal incumbents are opposed – not to mention the fact that Ontario's municipal incumbents are often so secure in their positions that they have been willing to endorse potentially disruptive institutional changes, such as online voting and major electoral reform (Anderson and Stephenson 2021, Goodman et al. 2024). Incumbent incentives alone cannot explain why other players in the municipal political game – challenger candidates, political parties, interest groups, even the provincial government with constitutional jurisdiction over municipal election law – have not encouraged or introduced municipal political parties.

In this article, we investigate an alternative explanation for the absence of municipal parties in Ontario: popular opposition. More specifically, we consider whether Ontarians want parties, why it is that they do or do not want parties, and who among them is more likely to support parties. Though it is politicians who ultimately decide whether or not to adopt local parties, they are wise to be conscious of public opinion. Accordingly, we developed three interrelated empirical studies that explore how Ontario's residents think about municipal partisanship. Using a large survey of the Ontario public (*N* = 4,038), carried out at the time of the 2022 Ontario municipal elections, we seek to answer three research questions. First, we explore the extent to which Ontarians’ attitudes about municipal political parties change when they think of those parties as either (a) appendages of the provincial or federal party system or (b) distinctive and municipality-specific parties. Using a survey experiment, we find that very little appetite among Ontarians for municipal parties regardless of their type.

In our second study, we explore the *reasons* that citizens provide for their attitudes for or against municipal political parties. Using systematic qualitative coding of thousands of open-ended survey responses (*N* = 2,364), we find that many citizens on both sides of the partisanship debate have sophisticated implicit theories of the character of municipal politics and the consequences of municipal parties for elections, policy-making, and inter-governmental relations. Those who prefer the independent-based status quo tend to emphasize the distinctiveness of municipal politics and policy, the value of independent thinking among non-partisan politicians, and the benefits of non-partisanship for local representation. Supporters of political parties, in contrast, emphasize the informational benefits of party affiliation for voters, along with the value of political parties for coherent and effective policy-making on municipal councils.

In our final study, we investigate the *types* of citizens who support or oppose municipal partisanship. Using thematic questions developed from the open-ended responses in study two, we created a new measure of “pro-partisan” or “pro-independent” attitudes. We explore how these attitudes are related to individuals’ ideological positions, partisanship, and local political interest. Our results suggest that while attitudes toward municipal parties do vary in ways that we would expect, the majority position even among more supportive subgroups (such as partisans or ideologues) is general opposition to municipal political parties.

Taken together, our analyses suggest that Ontario's citizens play an important role in the province's longstanding non-partisan equilibrium. While Ontarians are happy to incorporate partisan information into their municipal voting decisions when they have access to such information (McGregor Lucas and McGregor 2021), few wish to see political party competition in municipal elections. Given that citizens *do* use partisan information in their municipal vote choices when it is available, we suggest that there remains an opportunity for Ontario's municipal politics to shift into a very different – partisan – equilibrium. Given our findings, however, such a shift would probably originate in a dramatic exogenous shock or an act of high-risk political leadership.

## Local Political Parties in Canada

Political parties are regularly involved in municipal politics in many advanced democracies (Lightbody 2006). Municipal elections in the United Kingdom, a country with many political similarities with Canada, are highly partisan – in one Labour Party strategist's words, they function as “a sort of dummy run for some of the tactics and strategies [parties] might use for a national campaign.”^2^ In the United States, municipal elections are increasingly shaped by national policy debates and partisan animus between the Democratic and Republican Parties (Hopkins 2018). Municipal elections in The United States, Latin America, and Europe also regularly feature political parties and formal electoral slates.

In most Canadian provinces, local elections are non-partisan. In two provinces, however, parties of different types regularly contest municipal elections. In Quebec, electoral slates or “teams” linked to mayoral candidates (e.g., “Équipe Denis Coderre”) are very common, and more programmatic parties also occasionally find success (Quesnel-Ouellet, Belley, and Léveillée 1991; Sanger 2021). None of the parties that are active in Quebec's municipal elections have formal ties to existing federal or provincial parties, operating instead as distinct local parties that organize and contest elections only in their own municipality. Political parties are also well developed in British Columbia – especially Vancouver, where a well-developed municipal party system has existed for nearly a century (Armstrong and Lucas 2022; Cutler and Scott Matthews 2005; Tennant 1980). In British Columbia’s 2022 municipal elections, 53 municipal parties ran in 23 municipalities and school districts, 11 of which were in Vancouver. Only the Green Party of Vancouver has active ties to parties at other levels and only the right-wing populist “ParentsVoice BC” party organized across municipalities. For the most part, however, Canadian municipal elections are non-partisan, and Ontario fits the norm in this regard.

In contrast, Ontario's municipal elections are non-partisan. Ontario's *Municipal Elections Act* prohibits direct partisan involvement in campaigns and there are no mechanisms in place for candidates to officially designate themselves as a candidate of a political party (Ministry of Municipal Affairs and Housing 2017). While political parties have made occasional attempts to contest local elections in Toronto (Clarkson 1972; Filion 1999), and there is historical evidence of Labour Party involvement in local elections in Hamilton (Eidelman and Lucas 2023; Robin 1968), their presence has been minimal across Ontario for many decades.^3^ Despite a resurgence of locally-based advocacy organizations that develop policy and nominate candidates, such as Progress Toronto and Horizon Ottawa, there has been no sustained, concerted effort to reintroduce partisan politics in Ontario.

From a comparative perspective, this absence of political parties from Ontario's municipal politics is surprising – and, equally surprisingly, few have attempted to explain it. Some, as we noted earlier, point to limited incentives on the part of incumbent politicians to encourage political parties (Sayers and Lucas 2017); others merely note that non-partisanship is a historically entrenched aspect of municipal political culture in Ontario, one that is largely taken for granted (Good 2017; Sproule-Jones 2007). Yet these explanations are unsatisfactory. Comparative-historical analyses of party emergence in Canada and the United States have documented how incentives to create political parties can emerge both inside and outside a wide variety of legislative institutions (Aldrich 1995; Cox 1986), and political parties are extremely common in municipal governments in other advanced democracies, even in countries with similar electoral institutions (Copus 2004) and similar rates of incumbency advantage (Trounstine 2011, Warshaw 2019).

Moreover, even if incumbent politicians have little incentive to create political parties, *other* actors in the municipal political system may wish to initiate partisan teams. Municipal challenger candidates, facing the twin headwinds of incumbency advantage and low levels of voter information, may see considerable benefit in joining with candidates in other wards to develop shared policy promises, shared information tools and branding, and shared critiques of incumbent performance – a strategy that has proved successful in municipal campaigns earlier in Canada's history (Tennant 1980). Even more obviously, political parties that exist at the federal or provincial level may see some advantage in selecting candidates for office municipally. Local candidacy can provide political parties with additional opportunities to reward politically ambitious party loyalists, and can also serve as a training ground for candidates who aspire to enter provincial or federal politics later. Political parties can also benefit from the voter identification and mobilization efforts that occur during municipal elections – which create valuable up-to-date information on likely supporters in a particular community. And, as we noted above, local campaigns can give political parties the opportunity to “pilot” policy commitments or campaign messages for future elections at other levels.

Given the plausible incentives among challenger candidates and existing political parties to encourage or create municipal parties (along with similar incentives among interest groups and social movements, who may also wish to organize policy-focused slates), the absence of such parties in Canada's largest province is an important puzzle – one that cannot be satisfactorily explained by simply pointing to incumbent dominance or historical tradition. Here, we focus on another possible source of Ontario's non-partisan equilibrium: public opinion. Political actors who might otherwise see good reason to form municipal parties may shy away from doing so if they fear that the public will punish such efforts. This might create a stable equilibrium in which no one is willing to act as the first mover in the direction of a partisan municipal system. Moreover, political parties may be more difficult to initiate today, in an atmosphere of declining partisanship and decreasing trust in political parties (Clarke and Stewart 1998), than in earlier decades when political parties emerged in most other municipal political systems. Exploring this possibility requires that we systematically investigate ordinary citizens’ attitudes toward municipal parties in Ontario's current non-partisan setting.

## The Promise and Peril of Parties

Research on political parties and their effects on elections and representation have pointed to three main positive effects of political parties for municipal politics: turnout, information, and accountability. On the matter of turnout, Welch and Bledsoe (1986) observe that non-partisan municipal elections traditionally have lower voter turnout than those in which parties openly participate. This builds on the work of Lee (1960), who observed a substantially higher voter turnout rate in American cities with partisan elections than those with mandated non-partisanship. This difference in turnout may generally be a product of the work partisan groups put into mobilizing voters and ensuring their identified supporters participate in the electoral process (Karp 2012). Political parties provide accumulated expertise and knowledge to help candidates mobilize their supporters; in non-partisan systems, candidates must rely on their personal campaign team or their own knowledge of canvassing, advertising, messaging, and “get-out-the-vote” efforts, which may be limited. In the absence of partisan mobilization – and the activation of partisan identities associated with it – local participation may be more limited.

Political parties also affect the local information landscape. Municipal elections are often described as low-information events, particularly in a context of consolidated media and limited local news coverage. Political parties not only have an incentive to convey their policy commitments and “brand” to voters, but once established, they also provide voters with an easy heuristic with which to identify suitable candidates (Crowder-Meyer *et al*. 2020). By “producing a set of reference points for the individual elector,” Lightbody (2006, 231) notes, municipal political parties would offer “a bridge between a list of candidates and voters.”

Political parties may also improve political accountability after the election is over. Even the most engaged local residents often find it difficult to track their councillor's position on important votes in non-partisan councils, especially on larger councils. In the absence of a coherent policy agenda organized by a political party, political scientists since Adrian (1952) have worried that individual councillors could shift blame and avoid accountability when legislation performed poorly. In the absence of political parties, councillors may engage in strategic information disclosure to highlight their successes and downplay their failures (de Benedictis-Kessner 2021), strengthening incumbency advantage. Political parties have a clear incentive to provide information about council success (if in power) *and* council failure (if not in power), potentially easing the path for voters to reward or punish their representative's performance. In essence, the presence of an organized opposition (party) serves to keep the public better apprised of the business of council.

There are legitimate critiques of municipal partisanship that must be considered as well. One possibility is that a party system would remove the careful deliberation of council members from public view. Important decisions regarding policy may be made by partisan staff and enforced by a party whip, disallowing individual members from expressing their views (Lightbody 2006). Similar to this is the idea that parties can reduce the time for debate over issues and increase the time for debate over partisan “talking points.” Rather than focus on the important issues of governing, a municipal party driven by an institutionalized party apparatus, may attempt to win quick political victories over carefully considering the technical minutiae of urban systems.

Another possible drawback is the potential for further animosity to develop between levels of government. As municipalities are required to work closely the provincial governments in Canada, the possibility of each level maintaining a different partisan and/or ideological grounding could further strain the already complicated dynamics between province and municipality. When parties sought to contest the 1969 municipal election in Toronto, the province's Conservative Party raised this prospect and used it as justification for their non-participation in the race (Fowler & Goldrick, 1972)

We are also interested in understanding the *arguments* that citizens provide for and against municipal political parties. Understanding how ordinary citizens’ think about the likely consequences of municipal political parties – both good and bad – will help to clarify the possible role of public opinion in shaping the non-partisan structure of these non-partisan municipal elections. We focus particular attention on three interrelated questions: how much does public opinion on municipal political parties depend on the *type* of party under consideration? What reasons do citizens provide for their attitudes about municipal political parties? And what *types* of citizens are more or less likely to support municipal political parties in Ontario? Our aim is to provide, for the first time, an in-depth picture of attitudes on municipal partisanship in Ontario.

## Overview of Studies

To answer our research questions, we rely on data from a two-wave survey conducted at the time of the 2022 municipal elections in Ontario. The first wave was fielded just prior to the October 24 election (September 28 to October 20), and the post-election wave was fielded immediately after election day (October 25 to November 7). The survey was administered by Léger Research using participants from an existing online panel, with recruitment quotas for age, gender, and education. Surveys were completed online, via Qualtrics. Respondents were eligible to vote in the 2022 municipal elections (Canadian citizens, Ontario residents, and 18 or older). A total of 4,038 respondents completed the first wave, and 2,982 completed the second, for a return to sample rate of 73.4 percent.

While our research questions each capture one component of our larger focus on citizens’ attitudes toward municipal political parties, each question requires distinctive data and methods. We thus designed our project as three interrelated studies. We first use a survey experiment to explore citizens’ baseline levels of support for municipal parties, as well as the variation in this support when citizens are provided with information about federal/provincial versus specifically municipal parties. To better understand why Ontarians support or oppose the idea of municipal political parties, we then turn to a qualitative content analysis of open-ended follow-up questions to the survey experiment. This second study allows us to describe the rich variety of reasons that citizens provide in support of, or opposition to, parties in municipal politics. Finally, our third study explores the correlates of support or opposition to municipal political parties, using forced-choice statements drawn from the open-ended responses we collected in study two. By combining methods in this way – an experimental design in study one, qualitative content analysis in study two, and descriptive regression modeling in study three – we are able to provide a rich picture of citizens’ beliefs about municipal political parties, the kinds of citizens who are likely to support or oppose them, and, above all, citizens’ consistent overall opposition to the introduction of political parties at the municipal level in Ontario.

While our three studies are interrelated and mutually support one another, each involves distinct data, variables, and analysis. For this reason, we describe the relevant data, measures, and methods in each subsection below. We also provide complete wording for all survey questions in the supplementary material (Appendix I).

## Study 1: Party Type and Support for Municipal Parties

As noted earlier, participation in Canadian municipal elections by major federal or provincial parties, such as the Liberal Party or Conservative Party, is extremely rare in Canadian history. While such parties *do* occasionally seek municipal office – particularly smaller third parties, such as the New Democratic Party and the Green Party – municipal parties that are specific to one city are much more common. While the most common type of municipal election in Canada is non-partisan, when parties *do* form in Canadian municipalities, they are overwhelmingly of the distinctively municipal variety.

In our first study, we therefore seek to answer two questions: how much do citizens support the involvement of political parties in municipal politics, and how much does this support depend on the *type* of party that is involved? After all, each of these party types have specific advantages and disadvantages. City-specific parties might be well-suited to fit the unique needs of a particular city, unencumbered by the traditions and constraints of the federal or provincial party system – but distinctive municipal parties also require more effort on the part of voters, since their values and history are likely to be less clear in voters’ minds. On the other hand, federal or provincial political parties have a well-established reputation among voters, providing citizens with readily available cues about the candidates they should support (or avoid) – but they come at the cost of potentially importing irrelevant policy disagreements or partisan animosities from the federal or provincial levels into the municipal sphere. We therefore want to understand not only how citizens think about the presence of political parties in municipal politics, but also how these preferences shift when framed in terms of specifically municipal versus provincial/federal party involvement. Our data suggest that Ontarians have little appetite for municipal political parties regardless of the type of party under consideration.

### Data and Measures

To measure citizens’ preferences on municipal parties, we randomly assigned each respondent in our pre-election survey to answer one of three questions. The first group was asked about political parties in general, the second about political parties specific to a city, and the third about federal or provincial parties contesting local elections. The specific wording of the questions is as follows:

- [Control Group] In municipal elections in Ontario, candidates run as non-partisan independents. In many municipal elections outside Ontario, many candidates run as members of political parties. Do you think municipal candidates in Ontario should run as independents, or as members of parties?
- [Federal/Provincial Parties Treatment] In many municipal elections outside Ontario, many candidates run as members of political parties, such as the Liberals, Conservatives or NDP. Do you think municipal candidates in Ontario should run as independents, or as members of parties?
- [Municipal Parties Treatment] In municipal elections in Ontario, candidates run as non-partisan independents. In many municipal elections outside Ontario, many candidates run as members of unique municipal political parties, such as the “Civic Government Association” or the “Coalition of Progressive Electors.” Do you think municipal candidates in Ontario should run as independents, or as members of parties?

Response options for all three groups were identical: *Independents, Political Parties, and Don’t Know*. Because group assignment was random and our groups are well-balanced on relevant variables,^4^ a simple comparison of means allows us to test for any statistically significant differences in citizens’ preferences across the three groups.

### Results

**Figure 1.**
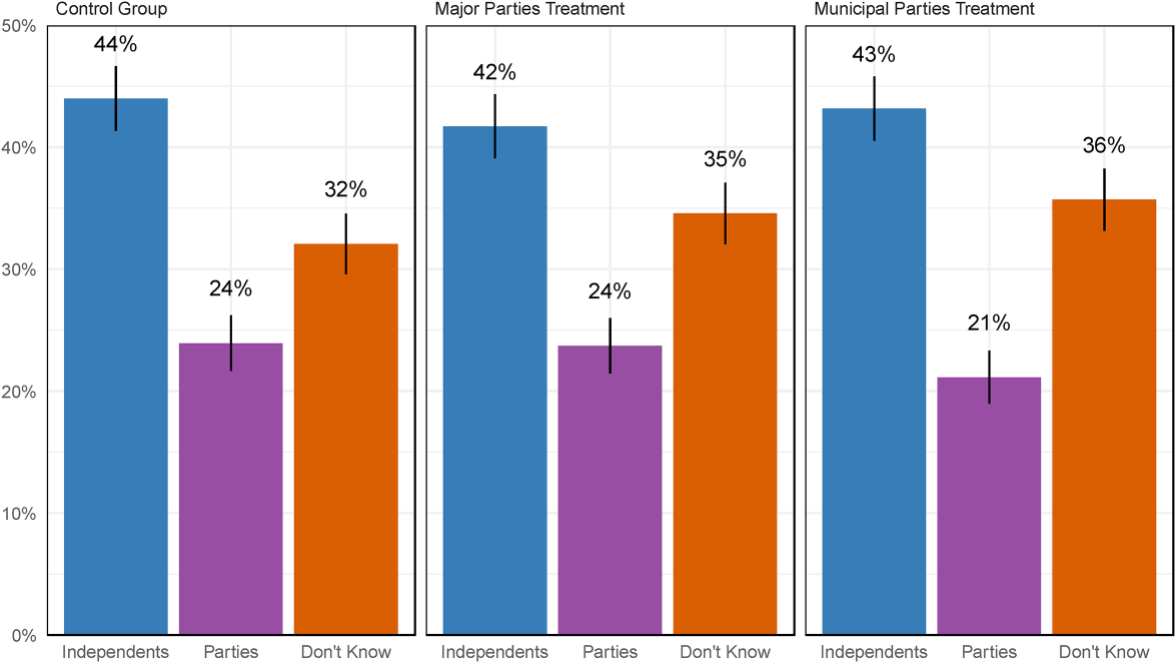
Support for local political parties – experimental results.

Figure 1 summarizes the distribution of responses in each of our three groups, with control group members in the left panel, the federal/provincial party treatment in the center, and the municipal party treatment on the right. The colored bars summarize the percentage of respondents who selected each option. Whiskers are 95 percent confidence intervals. Overall, survey respondents were consistent in their preference for the non-partisan status-quo. Pooling across all three groups, 43 percent of respondents preferred independent candidates, while just 23 percent preferred municipal political parties. More than a third (34 percent) had no opinion on the matter.^5^ In general, however, the pattern is clear: very few Ontarians would like to see political parties formally involved in their local elections.^6^

Interestingly, these attitudes do not meaningfully change when we specify the type of political party that might be involved. Across the three panels in Figure 1, the differences in those who choose “Independent,” “Parties,” or “Don’t Know” are substantively small and statistically insignificant.^7^ Ontarians’ lack of support for municipal political parties is not conditional on the type of party that might run for local office: they are just as unsupportive of distinctive municipal parties as they are of Liberals or Conservatives running for office.

It is also notable that across all three groups, “Don’t Know” was selected more often than “Political Parties.” This reinforces the impression that there is little appetite for municipal political parties – among those who *have* an opinion on the matter, it overwhelmingly favors independents. However, it also suggests that there may be some room for persuasion on this issue. From the standpoint of a supporter of political parties, one could perhaps conclude, most optimistically, that a majority of Ontarians are at least *not opposed* to the introduction of local political parties. Even so, there is no reason to suspect that those without an opinion on political parties would break strongly in favor of parties if they were provided with information and asked to develop an opinion on the matter – especially because, regardless of the information we provided in our experimental treatments, citizen attitudes were unmoved.

## Study 2: Citizens’ Beliefs about Municipal Political Parties

Our survey experiment revealed strong and consistent patterns of a lack of support for municipal political parties, regardless of the type of party under consideration. In our second study, we sought to understand the reasoning that lay behind these preferences – *why* citizens chose to support parties or independents in their responses to the survey experiment. In this study, we use a very large number of open-ended survey responses to understand the major themes that emerge when citizens are asked not only to express their preference for or against local parties, but also to explain their reasoning for that preference.

### Data and Measures

Immediately after the survey experiment in Study 1, we asked respondents an open-ended follow-up question about their reasoning:

- [Respondents who selected “independent” in the survey experiment] Why is that you think candidates should run as independents, rather than as members of parties?
- [Respondents who selected “party” in the survey experiment] Why is that you think candidates should run as members of parties, rather than as independents?

We received a total of 2,364 responses to these questions: 1,587 responses from pro-independent respondents and 777 responses from pro-partisan respondents.^8^ These responses offer a rich source of information about citizens’ attitudes concerning municipal political parties – to our knowledge, they are the first ever body of large-scale qualitative data of their kind.

We used an inductive thematic coding procedure to identify recurring themes in the open-ended responses to our questions. We began by selecting a random sample of 100 pro-independent and 100 pro-party responses. Research team members coded these sampled responses separately, and we then met to discuss the responses as a team to identify and discuss major themes, focusing on identifying and describing recurring arguments that citizens tend to make about the strengths or weaknesses of parties in municipal elections. Having developed this preliminary list of themes, one team member then proceeded through the full list of 2,364 responses, coding the appropriate theme for each response and, when necessary, adding additional themes to cover ideas and arguments that had not appeared in the initial sample. Following this full coding effort, we drew a second sample of 100 responses from the coded dataset, which we then discussed and validated as a team. While there was very minimal disagreement about the coding decisions, our final discussion led to some specific themes being combined into larger and more general arguments to simplify our presentation of results.

### Results

Our respondents offer a rich and diverse array of arguments both for and against municipal political parties. We summarize the most common arguments in Table 1; in each case, the table identifies the general theme and then provides a few examples from the open-ended responses themselves.

**Table 1.** Citizens’ Arguments for and Against Municipal Parties.

Argument	General theme	Examples
*Pro-Independent*	Municipal Distinctiveness	“Important to separate local politics from national and provincial ones.” “Don’t need party politics in municipal elections.” “Municipal is very different and party politics should not influence.”
	Independent Thinking	“It gives them a chance to formulate their own ideas and policies and not have to adhere to party policies.” “Prefer they think outside party policies.” “Their opinions can differ from the party.”
	Individual Merit	“Candidates should run on their own records and policies.” “I like the idea of voting for the individual instead of the party.” “Rather vote the person for their policies and not their ‘team.’”
	Focus of representation	“They should be representing the needs of the community without any political leaning.” “They should represent everyone no matter their political affiliation.” “So they can make decisions based on their area rather than following some party line.”
	General anti-partisan	“It is nice to escape partisanship.” “Less partisanship.” “Most parties are corrupt and self-serving.”
*Pro-Party*	Information (General)	“Gives a better sense of their overall outlook and philosophies.” “It gives you a better idea of what their views are.” “So you know their stance in spending and policies.”
	Information (Ideology)	“Easier to identify ideology.” “It makes it easier to know their political right or leftness.” “To know which way they lean.”
	Strength in Numbers	“It allows them to work in a team.” “There is more power to a group rather than an individual.” “Bigger groups make bigger impacts than individuals.”
	Get Things Done	“A bunch of independents with a variety of competing platforms and beliefs probably wouldn't ever get anything done.” “They have more potential of winning and making a difference.” “Won't be able to get much done if they aren't part of a party.”
	Accept Reality	“Because the independents heavily allude to their party affiliations anyways. Stop the charade.” “They are part of a party anyway, might as well let us know which one.” “Candidates follow party ideology anyway.”

The first pattern that the reader will notice that, regardless of theme, explicitly anti-party statements are common in the pro-independent statements. The opposite is not true of the pro-party explanations – they were also overwhelmingly focused upon parties, rather than independents. It is clear that views of parties heavily influenced attitudes over whether the independent-based statues quo should continue.

Among the pro-independent respondents, five themes were especially prominent. The most common argument concerned the distinctiveness of municipal politics and/or the need to protect municipal politics from provincial and federal interference. For these respondents, municipalities are fundamentally different from provincial or federal governments. However important political parties may be at other levels, these individuals insisted that they simply do not belong in municipal politics. One respondent stated that we simply “do not need an added layer of complexity to municipal politics.” Another said that they “don’t think [municipal candidates’] political views have anything to do with running a municipality.” The reasons that citizens provided for this distinctiveness were varied – some mentioned municipal policy issues, others insisted that municipal debates were not ideological, and many simply asserted that municipal government was different from other types of government. Whatever the exact source of the distinctiveness, however, these respondents agreed that political parties simply do not belong in *municipal* elections or policy making.

The next two common themes in the pro-independent responses – independent thinking and individual merit – both emphasize the value of freeing municipal candidates from the shackles of party discipline, party platforms, and party reputations. Those coded in the “independent thinking” theme tended to emphasize the need for local representatives who can think for themselves and deviate from party “doctrine.” In the closely related “individual merit” theme, the argument focused more directly on municipal elections: for these respondents, non-partisan politics allows each candidate to stand on their own platform or performance record, taking responsibility for their own policy promises and actions in office. Some respondents also emphasized that non-partisan politics would enable them to assess each candidate as an individual, rather than filtering their judgment through a partisan lens. “I vote for a known liberal councillor,” wrote one respondent, “and I am a conservative. I know her, would vote for her even if she was NDP, but bring in parties and then it is a different ballgame.”^9^

The fourth pro-independent theme, “focus of representation,” captures respondents who expressed deep distrust in partisan candidates’ ability to represent *all* constituents in the community, rather than merely focusing on fellow partisans. This view came in two general flavors. Some emphasized that partisan politicians owe their careers and loyalty to their parties, and thus could not be trusted to make decisions for the good of their constituents if those decisions conflicted with the party's interests. Others argued that partisan representatives would focus on the needs of like-minded partisans rather than the community as a whole. These “focus of representation” responses were especially striking because, given Canada's plurality electoral system, they arguably apply equally well to provincial and federal politics. Some respondents noted this fact explicitly; “candidates should represent the interests of those who elected them and not have any interference from a party machine,” one wrote, going on to say that “this also applies to other levels of government.” On this view, the absence of political parties ensures that elected representatives are motivated to represent *all* of the members of their community.

Finally, some respondents offered general anti-partisan sentiment as their justification for supporting non-partisan municipal politics. In this final theme, respondents generally made broad and negative comments about political parties, using words like “corrupt,” “cretins,” “sheep,” “destructive,” and so on. “Gotta draw the line somewhere,” wrote one respondent, in a characteristic example. “It's already too late for provincial and federal politics.” These respondents’ arguments were in some respects the opposite of the “municipal distinctiveness” theme – for this group, municipal parties were to be avoided not so much because municipal politics is distinctive, but rather because political parties are a bad idea at *all* levels of government.

Woven through these pro-independent responses – especially the “municipal distinctiveness” and “focus of representation” themes – was a recurring *localist* ethos, a sense that political parties might compromise municipal representatives’ focus on the needs of their specific municipalities. Some emphasized that party platforms developed to appeal to provincial or federal voting blocs might not be in the best interests of a single municipality, creating divided loyalties for partisan municipal politicians. Others emphasized that intergovernmental relations would suffer if municipal politicians belonged to the “wrong” partisan team: “I would not like to see party politics at the municipal level,” one wrote, “due to the conflict that might arise with federal and provincial parties.” This localist undercurrent captures citizens’ awareness of municipal governments’ role as *advocates* for their local community in a larger intergovernmental system (Eidelman and Lucas 2023) – a role that could be compromised if municipal politicians were attached to particular party groups.

Turning to the pro-party respondents, we find an equally rich and interesting mix of arguments. Among these responses, one theme stood above all: *information*. More than a third of those who support municipal parties emphasized that political parties would provide voters with valuable information about a candidate's priorities, policy commitments, and background. “There are too many candidates to remember who they all are and what they stand for,” one wrote. “If they’re a member of a party, it's easier to distinguish generally what they stand for.” This sentiment – that independent elections ask too much of voters who are pressed for time and confused by the indistinguishable mass of candidates – was very common.

Within this general emphasis on information, a substantial subset of pro-party respondents made specific reference to information about *ideological* commitments. As we noted earlier, past research on non-partisan municipal elections has suggested that many municipal voters do seek out information about local candidates’ party affiliations, and ideological proximity does influence vote choice in council and mayoral elections in Canada (Lucas and McGregor 2021; Lucas, McGregor, and Bridgman 2023). Many pro-party respondents made this connection explicit: “if I am a conservative person,” one wrote, “I should know if the person running is Liberal or other party which goes against my values.” Another wrote that party affiliation would make it “easier to identify their political ideology.” As Canadian municipal elections researchers have suggested, many citizens use information about party affiliation as an important clue for understanding the ideological position of municipal election candidates.

While information was the most common theme among pro-party respondents, a substantial group emphasized political parties’ capacity to get things done and the advantages of working together as a team. Those in the “*strength in numbers*” theme made this argument in general terms, highlighting the advantages of teamwork rather than individual effort. In the “*get things done*” theme, respondents were more specific about the advantages they had in mind: enacting policies that would benefit local residents. One respondent in Toronto noted that “policy making is slowed by having all independent councillors.” Another made reference to their experience elsewhere in Canada. “This gives coherence to the work of council and more gets done. I lived in Montreal for [anonymized] years and appreciated the party system.”

A final prominent theme among pro-party respondents was the idea that municipal politics is already partisan. These respondents argued that most candidates who run for municipal office have some affiliation with a political party, and it would be valuable to bring these affiliations out into the open. “The voting pattern of municipal councillors makes it obvious what party they represent,” one respondent wrote. “By running as members of a political party, they are being more open and honest. That way voters will be voting for candidates who share their values and concerns and priorities.” On this view, municipal elections would be clearer for voters and more honest and transparent if candidates’ party affiliations were brought out into the open, rather than remaining hidden in the shadows and known only to insiders.

Overall, we found that ordinary citizens possess remarkably rich and detailed theories of the advantages or disadvantages of municipal parties. While these well-developed responses are by no means universal – readers should recall that about a third of the survey experiment respondents selected “Don’t Know” and were excluded from the follow-up open-ended question – it is nevertheless true that a majority of survey respondents were able to provide a clear reason for pro-independent or pro-party preference. Their responses capture a wide range of important and competing arguments, including many prominent areas of debate in political science research on local and urban politics – such as the informational benefits of party affiliation for lower-information voters. The fact that most citizens have well-developed arguments for their preferences on municipal political parties suggests that they are unlikely to quickly change their minds about the benefits and drawbacks of local political parties.

## Study 3: Who Supports Municipal Political Parties?

Thus far, our results suggest that Ontarians are generally unsupportive of municipal political parties regardless of the type of party under consideration, and that many citizens offer clear reasons for their pro-party or anti-party sentiment. However, we expect that these attitudes will also vary in theoretically important ways: that is, some types of citizens will be more likely to support municipal parties than others. In our final study, we explore the correlates of support for municipal parties, focusing particular attention on four theoretically salient variables: partisanship, ideology, political attention, and views of municipal turnout.

We expected that *partisans* – those who feel a meaningful connection to a provincial political party in Ontario – would be more likely than non-partisans to support municipal parties. Past research has shown that partisans are more likely to look for information about the party affiliation of ostensibly non-partisan municipal candidates (Lucas and Santos 2021, Stephenson et al. 2018), which suggests that many partisans would be happy to have access to partisan information in municipal contests.

We also expected that *ideologues* – those who have extreme rather than moderate ideological positions – would also be more supportive of municipal parties. Ideological extremists are more likely to simplify the world into easily distinguishable choices (Lammers et al. 2017; Prooijen and Krouwel 2019). At the poles of the ideological spectrum, ideologues do not experience the same tension when trying to develop vote choices or develop partisan attachments. Given the emphasis in our open-ended responses on the specifically *ideological* signals that municipal party affiliations would provide, along with the important role of ideology in vote choice in Canadian municipalities, we expected that ideological extremists would be more likely to see value in municipal political parties.

Because party affiliations provide a simple heuristic for voters with low information about individual candidates, we also expected that citizens who pay close attention to municipal politics would be more skeptical about parties than citizens who pay little attention. High-attention citizens are more likely to be willing to do the hard work of researching each independent candidate's policy commitments and past experience (Moore, McGregor, and Stephenson 2017). Low-attention citizens, in contrast, may want to vote in a municipal election but have insufficient information with which to make their decisions. If this is so, support for municipal political parties might decline as attention to municipal politics increases.

Finally, and more speculatively, we were interested in understanding if citizens’ attitudes on *municipal turnout* would be associated with support for municipal political parties. Past research has suggested that partisan elections are associated with higher turnout (Carel 2007), and many of our pro-party respondents noted that party affiliations would make the voting process easier by providing clear information about candidates. While only a small number of respondents explicitly mentioned turnout in their open-ended responses, we nevertheless explore the possibility that preferences for higher municipal turnout are associated with a desire for partisan local elections.

### Data and Measures

Our third study relies on a set of forced-choice questions about municipal political parties that we included in the post-election wave of our 2022 Ontario municipal election survey. We developed these questions from the arguments that citizens had themselves offered in their open-ended responses in the pre-election survey. Drawing on the themes discussed above, we developed and fielded three pro-partisan and three anti-partisan questions, to which participants responded on a four-point agree/disagree scale:

- *Focus of representation.* Municipal candidates should continue to run as independents, because political parties put their own interests ahead of the interests of their constituents.
- *Independent thinking.* Municipal candidates should continue to run as independents, because independent candidates can take their own positions, rather than simply adopting the positions of their parties.
- *Individual merits*. I would prefer to base my decisions on the characteristics of local candidates, rather than the political party they belong to.
- *Getting things done.* Municipal candidates should run as members of parties, because political parties are better at getting things done than independent councillors.
- *Ideological information*. If we had political parties in municipal elections, it would be easier to know where candidates stand in terms of their “left,” “center,” or “right” position on the ideological spectrum.
- *Accept reality*. “Most people who run for municipal office are affiliated with political parties, so voters might as well know about these party affiliations when making decisions about who to support.”

These questions were meant to capture themes in the open-ended responses that were common and thus likely to be readily understandable to respondents, while also encapsulating diverse reasons for and against local parties. To measure the correlates of citizens’ overall support or opposition to municipal parties, we combined the six questions into an overall “pro-partisan” scale (reversing the direction of the coding for the three anti-partisan questions).^10^ This “pro-partisan” scale is our main outcome variable of interest in this analysis.

To measure *partisanship*, we follow standard practice among Canadian political scientists and define partisans as any individual who identifies “somewhat” or “very” strongly with a political party. We measure *ideology* with a standard 0–10 left-right self-placement scale. We measure *attention* with a survey question about the respondent's self-reported level of attention to the municipal election on a 0–10 scale. Finally, we measure each respondent's preference on municipal turnout with a question asking each respondent about the lowest level of turnout in a municipal election that they would find acceptable, ranging from 0 percent to 100 percent. We provide complete wording for each of these questions Appendix I.

In our main analysis, we report relationships for each variable using non-parametric locally weighted smoothing. However, we supplement these findings with bivariate and multiple variable linear regression results in Appendix III.

### Results

**Figure 2.**
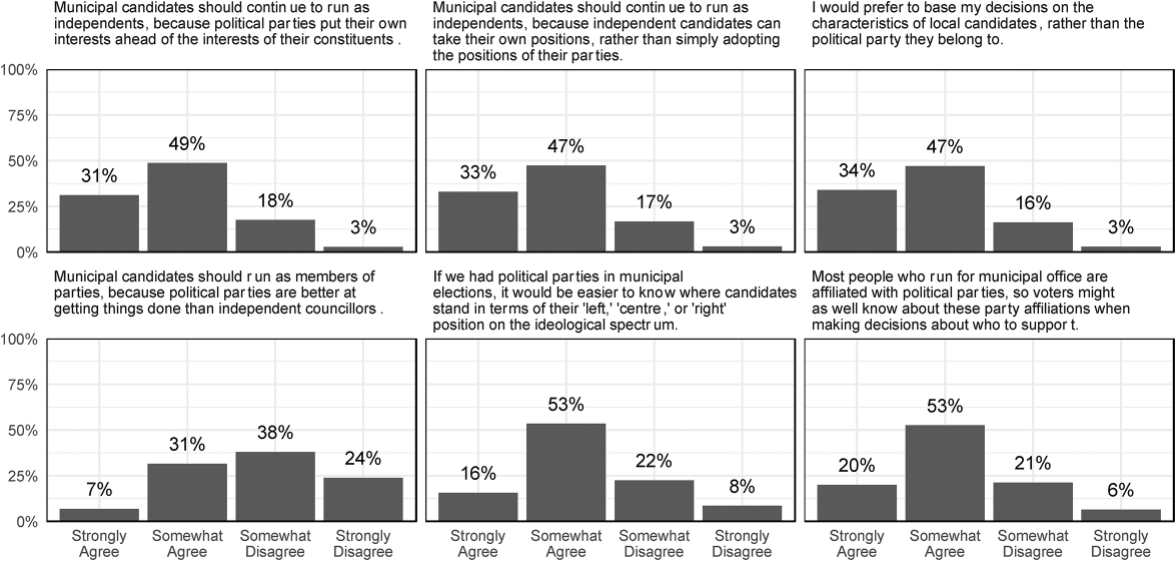
Attitudes toward parties: descriptive overview.

**Figure 3.**
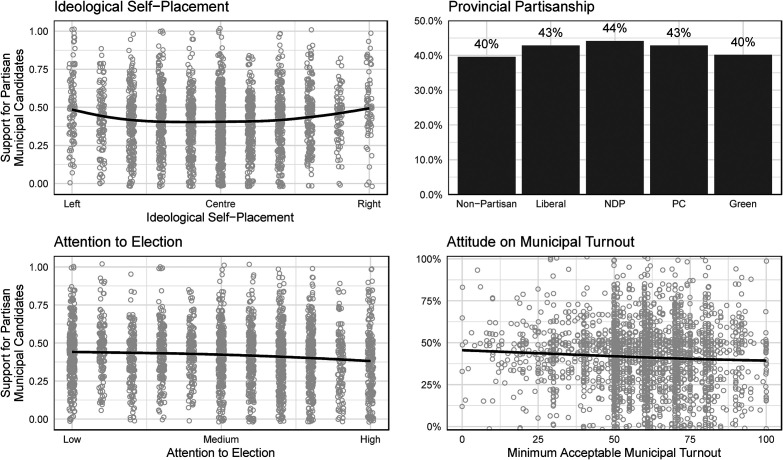
Attitudes toward parties: correlates.

We summarize the results of our first study in Figures 2 and 3. Figure 2 provides a descriptive overview of responses to our three anti-party statements (first row) and pro-party statements (second row). In Figure 3, we summarize the relationship between respondents’ overall pro-party sentiment, summarized in the vertical axis, and their ideological self-placement, provincial party identification, attention to the municipal election, and attitude concerning the minimum acceptable level of turnout in municipal elections.

Our results reinforce yet again the dominance of anti-party sentiment among citizens. Clear majorities somewhat or strongly agree with the three anti-party statements at the top of figure 2: 80 percent agree that independent candidates focus on the interests of their constituents, 80 percent agree that independent candidates can avoid unnecessary party discipline, and 81 percent agree that they prefer to make vote choices on the basis of individual candidates rather than political parties. In the second row, responses are more mixed: while majorities agree that municipal parties would make it easier to determine a candidate's ideology and acknowledge that many municipal candidates *do* have party affiliations, only 38 percent agree that municipal parties would be better at “getting things done” than independents. These results confirm that most citizens are opposed to formal party involvement in municipal politics: the average score on the 0–18 pro-party scale was 7.5.

Figure 3 suggests that this anti-party sentiment is quite consistent among the public. In each panel, the vertical axis summarizes citizens’ expected scores on a pro-party scale that ranges from zero (extreme opposition to parties in municipal politics) to eighteen (extreme support); in this scale, values above nine indicate overall support, and values below nine indicate opposition. What is most clear across all four panels is the widespread *opposition* to political parties, with expected values below nine regardless of ideology, partisanship, attention, and turnout attitudes.

Despite this widespread opposition, pro-party attitudes *do* vary across citizens. As expected, ideologues of both the left and the right are modestly more supportive of political parties in municipal politics than are citizens who place themselves in the ideological center (*p* < 0.01). Similarly, those who identify with the major political parties in Ontario – the Progressive Conservatives, New Democratic Party, and Liberal Party – are more supportive of municipal party politics than are non-partisans and Greens.^11^ Overall, strong ideologues and partisans are modestly more supportive of municipal political parties than their ideologically moderate or non-partisan counterparts – yet, even fierce ideologues and strong partisans remain skeptical about local political parties.

The final panels in Figure 3 – attention and turnout – also reveal modest but statistically significant relationships. Those who paid more attention to their local mayoral election are slightly less supportive of municipal political parties (*p* < 0.01), perhaps reflecting the fact that these high-attention voters already feel that they have the information they need to cast an informed vote. For turnout, however, the relationship is the opposite to what we expected – those who believe that higher turnout rates are necessary for legitimate municipal elections are slightly *less* supportive of municipal political parties (*p* < 0.01). Individuals who believe that municipal turnout needs to be much higher than is typical in Ontario do not appear to believe that parties would provide a valuable means by which to boost local turnout.

Despite their statistical significance, all of the relationships we have uncovered are substantively modest: comparing ideological extremists to moderates, for instance, we find an expected difference of just 1.4 points on the eighteen-point pro-partisanship scale. While some citizens – such as ideologues and partisans – are likely to be more receptive than others to the idea of municipal political parties in Ontario, majorities in all of these groups remain skeptical about political parties in municipal politics.

## Discussion

Our goal in this article has been to investigate an often overlooked explanation for the absence of political parties in municipal politics: popular opposition. More specifically, we have explored whether Ontarians want parties, why it is that they do or do not want parties, and who among them is more likely to support parties. Though it is politicians who ultimately decide whether or not to adopt local parties, they are wise to be conscious of public opinion. Ontarians appear satisfied with the non-partisan status quo municipally, despite the presence of party systems both provincially and nationally. We suspect that these public attitudes help to explain the continued, and relatively uncommon, absence of non-partisanship at the local level.

Ontario stands out among advanced democracies in the depth and consistency of its commitment to municipal non-partisanship. In this study, we have explored the basis for this commitment in public opinion among residents themselves. We found, first, that few Ontarians support party involvement in municipal politics, regardless of the type of party under consideration. We also found that, while about a third of Ontarians have no opinion on the issue of municipal political parties, those who *do* have an opinion on the matter are generally able to provide persuasive arguments for that opinion. Among pro-independent or anti-partisan citizens, arguments about the distinctiveness of municipal politics and the need for independent thinking on city councils predominate. Among those who support political parties, opportunities for clear informational cues, and the ability to work together as a team to get things done, were the most common arguments.

Drawing on the rich arguments we found in our open-ended responses in study two, our third study developed a measure of pro-partisan or anti-partisan sentiment. Unsurprisingly, given our earlier findings, this measure confirmed that most Ontarians were strongly opposed to municipal political parties. Ideologues, partisans, and those with lower levels of interest in municipal politics are, on average, more supportive of municipal political parties than are moderates, non-partisans, and high-interest citizens, but these differences are substantively modest. Across nearly all relevant subgroups of the public, anti-partisan sentiment predominates.

Once again, our results suggest that Ontario's non-partisan equilibrium has a strong grounding in public sentiment. In our informal conversations with municipal candidates and campaign managers in Canada, we have often heard that candidates are unwilling to organize themselves into parties or slates for worry that voters will punish them for having done so. Our results suggest that these worries have merit. While many voters are eager to *have* information about candidates’ party affiliation and ideological position (Eidelman and Lucas 2023), and they *use* such information to inform their voting decisions when they do have it (Lucas, McGregor, and Bridgman 2023), it is nevertheless true, given our results, that most Ontarians prefer the non-partisan status quo.

What remains to be seen is whether our findings travel to other municipal contexts. Ontario is one of eight Canadian provinces where local elections are non-partisan, and future work should consider whether our findings hold in those provinces. Canadian provinces where parties *are* present – B.C. and Quebec – could be considered in a similar manner to see if electors prefer the partisan status quo, or if they would choose to shift to a non-partisan system. Ontario is an important case, but others inside and outside Canada must be considered in order for us to more fully understand the views of citizens toward partisanship at the municipal level.

Whatever merits municipal parties may have, then, anyone who wishes to encourage them in Ontario's municipal politics will do so in the face of a skeptical public. Still, for those who are advocates of municipal parties, the substantial percentage of “don’t know” responses in our survey experiment may represent a glimmer of hope. Perhaps some of these undecided Ontarians could be persuaded about the value of local parties. But among Ontarians who do hold an opinion on the matter, there is a strong preference for the non-partisan status quo. Even if the Ontario provincial government were to change the rules to incentivize local parties, municipal candidates might still insist on running as independents, fearing that local voters would punish them for introducing parties into the local arena.

We suspect that many municipal contexts in which non-partisanship predominates are likely to face a similar version of the paradox we describe here: voters who are keen to *use* partisan information in municipal voting decisions but who dislike the idea of introducing political parties into municipal politics. In this situation, it is certainly possible to imagine a counterfactual scenario – a scenario that is not uncommon in other countries – in which voters remain skeptical about the value of political parties but grudgingly accept a very different municipal status quo in which parties are regularly involved. To move to that different equilibrium, however, would probably require one of two interventions. A substantial exogenous shock – such as an intervention by the provincial government, which has jurisdiction over municipal election law, strongly incentivizing candidates to form themselves into like-minded slates or parties – might shift municipal politics in a partisan direction while allowing local candidates to avoid some of the blame for having done so. Alternatively, an act of high-risk political leadership by a municipal candidate, to form a coherent new political party or to link themselves explicitly to a provincial or federal party, might, if successful, result in a shift to a new partisan equilibrium. Such a move was attempted in Ontario in the 1960s, and its failure has served as a cautionary tale in the decades that followed (Clarkson 1972). Were it to be attempted by a successful candidate, however – such as an incumbent mayor of a major city – the results might yield a very different lesson. Either way, the move would involve substantial risk, in the face of an electorate that, we have found, is resolutely opposed to municipal partisanship.

## Supplemental Material

sj-docx-1-uar-10.1177_10780874231224707 - Supplemental material for Understanding Support for Municipal Political Parties: Evidence from Canada Supplemental material, sj-docx-1-uar-10.1177_10780874231224707 for Understanding Support for Municipal Political Parties: Evidence from Canada by Michael McGregor, Jack Lucas, Chris Erl and Cameron D. Anderson in Urban Affairs Review

## Notes

1. A record low of 36.3% of eligible voters cast a ballot in Ontario’s 2022 municipal elections. This beat the previous record low of 38.3%, set in 2018. Of the 2,842 seats available across Ontario, 548 were acclaimed, including 139 Heads of Council (Mayors or “Reeves” in smaller municipalities). A record eight percent of all municipalities saw entire councils acclaimed (AMO 2022).

2. Alastair Campbell, *The Rest is Politics Podcast*, April 11, 2023.

3. During Toronto’s municipal elections in 1969, both the left-wing New Democratic Party (NDP), and the Liberal Party formally participated, nominating candidates for school trustees, councillors, and, in the case of the Liberals, for mayor (Clarkson 1972; Filion 1999). Jack Layton, running under the banner of The NDP made a failed attempt to run for mayor in 1991.

4. A balance test (see Appendix II) reveals that the groups are balanced on the basis of age, gender, education, ideology, attentiveness, and attitudes towards turnout, and nearly balanced on partisanship (there is a slight imbalance in the rate of “Green” and “NDP” partisans between some groups). We re-ran the analysis while controlling for partisanship, and the substantive conclusions remain unchanged, indicating that this minor imbalance has not affected the null experimental findings.

5. We suspect that the size of the “don’t know” group might be larger in the general population. Respondents to our opt-in survey were recruited to answer questions on the topic of politics, and we suspect that the sample is more knowledgeable about politics than the general population.

6. This finding is consistent with recent research from the U.S., by Crawford (2022), who uses Cooperative Election Study to demonstrate that Americans too have a preference for non-partisan local elections.

7. Chi^2^ tests are not statistically significant when comparing group 1 and group 2 (*p*=0.35), group 1 and group 3 (*p*=0.08), and group 2 and group 3 (*p*=0.28).

8. Those who selected “Don’t Know” in the survey experiment were not asked the follow-up question. Pro-independent supporters were slightly more likely than pro-party supporters to provide a response to the open-ended question: 91% of pro-independent supporters completed the question, compared to 84% of pro-party respondents.

9. We have corrected two minor typos in this response.

10. This scale is reliable (alpha = 0.79) and captures a meaningful unidimensional latent quantity (in a factor analysis of the six items, the Eigenvalue for the first factor is 2.48, with all further factors below 1.0).

11. The difference between PC/NDP/Liberal partisans and non-partisans is statistically significant (*p*<0.01). Green partisans are not more pro-municipal-party than non-partisans.
