# Supplementary material for: Understanding Support for Municipal Political Parties: Evidence from Canada
Source: Urban Aff Rev Thousand Oaks Calif. 2024 Jan 15;60(5):1382–410. doi: 10.1177/10780874231224707 (PMC11315630; doi:10.1177/10780874231224707)
Supplement: sj-docx-1-uar-10.1177_10780874231224707 - Supplemental material for Understanding Support for Municipal Political Parties: Evidence from Canada [file sj-docx-1-uar-10.1177_10780874231224707.docx]

**Online Appendices**

**Appendix I: Survey questions**

Party or independents (For studies 1 and 2)

*In municipal elections in Ontario, candidates run as non-partisan independents. In many municipal elections outside Ontario, many candidates run as [experimental text here]. Do you think municipal candidates in Ontario should run as independents, or as members of parties?*

- Independents
- Parties
- Don’t know/prefer not to say

*[Control Group text] members of political parties.*

*[Federal/Provincial Parties Treatment] members of political parties, such as the Liberals, Conservatives or NDP.*

*[Municipal Parties Treatment] members of unique municipal political parties, such as the “Civic Government Association” or the “Coalition of Progressive Electors.”*

[If replied to above question with “Independents”]: *Why is it that you think candidates should run as independents, rather than as members of parties?*

- [Open ended box]

[If replied to above question with “Parties”]: *Why is it that you think candidates should run as members of parties, rather than independents?*

- [Open ended box]

Control variables (for balance test in study 1)

*In what year were you born?*

- Drop down menu with years provided

*Are you…?*

- A man
- A woman
- Non-binary
- Another gender, please specify:

*What is the highest level of education that you have completed?*

- No schooling
- Some elementary school
- Completed elementary school
- Some secondary/ high school
- Completed secondary/ high school
- Some technical, community college, CEGEP, College Classique
- Completed technical, community college, CEGEP, College Classique
- Some university
- Bachelor's degree
- Master's degree
- Professional degree or doctorate

Attitudes towards parties (study 3)

*Municipal candidates should run as members of parties, because political parties are better at getting things done than independent councillors.*

- Strongly disagree
- Somewhat disagree
- Somewhat agree
- Strongly agree

*If we had political parties in municipal elections, it would be easier to know where candidates stand in terms of their "left," "centre," or "right" position on the ideological spectrum.*

- Strongly disagree
- Somewhat disagree
- Somewhat agree
- Strongly agree

*Most people who run for municipal office are affiliated with political parties, so voters might as well know about these party affiliations when making decisions about who to support.*

- Strongly disagree
- Somewhat disagree
- Somewhat agree
- Strongly agree

*Municipal candidates should continue to run as independents, because political parties put their own interests ahead of the interests of their constituents.*

- Strongly disagree
- Somewhat disagree
- Somewhat agree
- Strongly agree

*Municipal candidates should continue to run as independents, because independent candidates can take their own positions, rather than simply adopting the positions of their parties.*

- Strongly disagree
- Somewhat disagree
- Somewhat agree
- Strongly agree

*I would prefer to base my decisions on the characteristics of local candidates, rather than the political party they belong to.*

- Strongly disagree
- Somewhat disagree
- Somewhat agree
- Strongly agree

Explanatory variables (for study 3)

*In politics, people sometimes talk of left and right. Where would you place yourself on this scale?*

- Slider provided ranging from 0 (left) to 10 (right)

*In provincial politics, do you usually think of yourself as a:*

- Liberal
- NDP
- Progressive Conservative
- Green
- Another party
- None of these

[If selected a party in previous question] *How strongly [party name] do you feel?*

- Very strongly
- Fairly strongly
- Not very strongly

*On a scale from 0-10, how much attention did you pay to the mayoral election campaign?*

- 0 (No attention)
- 1
- …
- 9
- 10 (A lot)

*In your opinion, what is the lowest level of turnout in municipal elections that is acceptable?*

- Slider provided that ranges from 0 to 100%

**Appendix II: Experimental balance test**

The following table shows multi-nominal logistic regression results, with the experimental treatment groups serving as the outcome variable. Explanatory variables include age (in years), gender, education (university educated versus other), and the same variables included in Figure 1 in study 1 ideology, partisanship (baseline = non-partisan), attentiveness, and opinions on the appropriate level of turnout. With the exception of age, all variables are coded to range from 0 to 1.

|  | Group 2 vs. Group 1 | Group 3 vs. Group 1 | Group 3 vs. Group 2 |
| --- | --- | --- | --- |
| Age | 0.00 (0.00) | 0.00 (0.00) | 0.00 (0.00) |
| Woman | -0.05 (0.11) | -0.02 (0.11) | 0.03 (0.11) |
| University education | 0.17 (0.11) | 0.13 (0.11) | -0.04 (0.11) |
| Ideology | 0.37 (0.27) | 0.17 (0.27) | -0.19 (0.27) |
| Conservative | -0.01 (0.16) | 0.13 (0.16) | 0.15 (0.17) |
| NDP | 0.41 (0.17)* | 0.45 (0.17)** | 0.05 (0.17) |
| Liberal | 0.21 (0.16) | 0.26 (0.16) | 0.05 (0.16) |
| Green | 0.05 (0.29) | 0.55 (0.27)* | 0.50 (0.28) |
| Attentiveness | -0.21 (0.18) | 0.00 (0.18) | 0.21 (0.18) |
| Appropriate level of turnout | 0.14 (0.30) | 0.15 (0.31) | 0.00 (0.31) |
| Constant | -0.45 (0.31) | -0.72 (0.32)* | -0.27 (0.32) |
| N | 2,194 | | |
| Pseudo R2 | 0.004 | | |

Entries report coefficients and standard errors.
* p < 0.05, ** p < 0.01

**Appendix III: Additional Detail and Tables – Study 1**

The table below summarizes OLS models of the bivariate relationship between each of our independent variables of interest and overall pro-party attitudes (columns 1-4), along with a model including all four variables (column 5). All independent variables have been rescaled to range between zero and one; the resulting coefficients can thus be interpreted as the expected difference in pro-party attitudes (on the 0-18 scale) when comparing individuals at the minimum and maximum values of each variable. For party identification, the base category is non-partisans.

|  | **Bivariate 1** | **Bivariate 2** | **Bivariate 3** | **Bivariate 4** | **All Vars** |
| --- | --- | --- | --- | --- | --- |
| Ideologue | 1.213*** |  |  |  | 1.347*** |
|  | (0.217) |  |  |  | (0.249) |
| Attention |  | -1.042*** |  |  | -1.202*** |
|  |  | (0.200) |  |  | (0.226) |
| Turnout Preference |  |  | -1.309*** |  | -1.499*** |
|  |  |  | (0.384) |  | (0.388) |
| Liberal Partisan |  |  |  | 0.591*** | 0.710*** |
|  |  |  |  | (0.175) | (0.193) |
| NDP Partisan |  |  |  | 0.824*** | 0.816*** |
|  |  |  |  | (0.181) | (0.202) |
| PC Partisan |  |  |  | 0.587*** | 0.592*** |
|  |  |  |  | (0.166) | (0.189) |
| Green Partisan |  |  |  | 0.102 | -0.010 |
|  |  |  |  | (0.343) | (0.368) |
| Num.Obs. | 2929 | 2977 | 2645 | 2928 | 2563 |
| * p < 0.1, ** p < 0.05, *** p < 0.01 | |  |  |  |  |
